# Regulatory roles of HSPA6 in *Actinidia chinensis* Planch. root extract (acRoots)‐inhibited lung cancer proliferation

**DOI:** 10.1002/ctm2.46

**Published:** 2020-06-05

**Authors:** Lingyan Wang, Jiayun Hou, Jianxin Wang, Zhenghua Zhu, Wei Zhang, Xuemei Zhang, Hui Shen, Xiangdong Wang

**Affiliations:** ^1^ Zhongshan Hospital Institute of Clinical Science Shanghai Medical College, Fudan University Shanghai China; ^2^ Department of Pharmaceutics, School of Pharmacy Shanghai Medical College Fudan University Shanghai China; ^3^ Department of Respiratory, Zhongshan Hospital Shanghai Medical College Fudan University Shanghai China; ^4^ Department of Physiology and Pathophysiology, School of Basic Medical Sciences Shanghai Medical College Fudan University Shanghai China; ^5^ Center for Tumor Diagnosis and Therapy Jinshan Hospital Fudan University Shanghai China

**Keywords:** acRoots, drug sensitivity, HSPA6, lung cancer, p53

## Abstract

*Actinidia chinensis* Planch. root extract (acRoots) as one of Chinese traditional medications has been applied for antitumor therapy for decades, although the exact mechanisms have not been revealed. Our present study aimed to define the inhibitory specificity and pattern of acRoots in the lung cancer cell lines by comparing 40 types of cancer cell lines, select acRoots‐associated inflammation target genes from transcriptional profiles of acRoots‐sensitive and less‐sensitive lung cancer cell lines, and validate the correlation of acRoots‐associated inflammation target genes with prognosis of patients with lung cancer. We selected acRoots‐sensitive (H1299) and less‐sensitive lung cancer cells (H460) and found that the sensitivity was associated with the appearance of p53. The heat shock 70 kDa protein 6 (HSPA6) was defined as a critical factor in regulating cell sensitivity probably through the interaction with intra‐HSPA family members, inter‐HSP family members, and other families. The degree of cell sensitivity to acRoots increased in both sensitive and less‐sensitive cells after deletion of HSPA6 genes. Thus, our data indicate that HSPA6 and HSPA6‐dominated molecular network can be an alternative to modify cell sensitivity to drugs.

## Graphical abstract

acRoots inhibited growth of multicancers and had related specificity and pattern of lung cancer cells. HSPA6 and networks regulated sensitivity of lung cancer cells to acRoots associated with the appearance of p53. HSPA6 signal pathways can be an alternative to modify cell sensitivity to drugs.

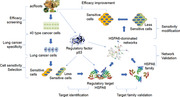

**Abbreviations**
acRoots: *Actinidia chinensis* Planch. root extract
ATF2: activating transcription factor 2
EP3: prostaglandin E receptor 3
HCC: hepatocellular carcinoma
HGS: human glutamine synthetase
HRG: histidine‐rich glycoprotein
HSPA6: heat shock 70 kDa protein 6
MMP2: matrix metallopeptidase 2
OASL: 2′‐5′‐oligoadenylate synthetase like
PI3K: phosphatidylinositol 3‐kinase
PCSK9: proprotein convertase subtilisin/kexin type 9
PSC: pancreatic stellate cells
PTGES: prostaglandin E synthase

## 1 INTRODUCTION

*Actinidia chinensis* Planch. root extract (acRoots) as one of Chinese traditional medications has been applied for antitumor therapy for decades. The interaction between acRoots and cancer cells is dependent upon biological types, origins, malignancies, and stages of cancers. Preclinical evidence showed that acRoots could inhibit the growth of lung cancer cell and increase its apoptosis by altering immune‐associated gene profiles via the phosphatidylinositol 3‐kinase (PI3K)‐2′‐5′‐oligoadenylate synthetase like (OASL) signal pathway.^1^ OASL may play a crucial role in adjusting the sensitivity of lung cancer cell and development of drug resistance to acRoots. It was proposed that acRoots may change biological activities of decisive regulators or checkpoints as well as associated signal pathways, leading to alternations of cell sensitivity and resistance to drug. This will be an alternative to refresh those cancer cells that become resistant during drug therapies.

In addition, acRoots was found to have direct inhibitory effects on cancer cell proliferation, movement, invasion, and metastasis in many cancers. For example, the inhibitory roles of acRoots hepatocellular carcinoma (HCC) cell growth depend upon the degree of malignancy, stages of cell cycle, and doses, probably through altering metabolic signaling responses and cancer cell inflammation gene clusters such as prostaglandin E receptor 3 (EP3) or proprotein convertase subtilisin/kexin type 9 (PCSK9).^2^, ^3^ AcRoots could downregulate gene and protein expression of those key regulators, production matrix metallopeptidase 2 (MMP2), the vascular endothelial growth factor, matrix metallopeptidase 9, and epidermal growth factor receptor, and capacity of cholesterol synthesis and uptake, for example, intracellular cholesterol levels and 3,3′‐dioctadecylindocarbocyanine‐labeled low‐density lipoprotein.

The present study aimed to define the inhibitory specificity and pattern of acRoots in lung cancer cells by comparing 40 types of cancer cells, select acRoots‐associated inflammation target genes from transcriptional profiles of acRoots‐sensitive and less‐sensitive lung cancer cells, and validate the correlation of acRoots‐associated inflammation target genes with prognosis of lung cancer patients. From screening, identification, and validation, we selected heat shock 70 kDa protein 6 (HSPA6) to be an inflammation target and furthermore investigated gene expression of 12 heat shock protein family members after treatment with different doses of acRoots and 15 elements within HSPA6‐dominated molecular networks. Furthermore, we evaluated the decisive role of HSPA6 in the sensitivity of lung cancer cell to acRoots treatment by monitoring the balance of cell proliferation and apoptosis of acRoots‐sensitive and less‐sensitive lung cancer cells with or without HSPA gene.

## 2 MATERIALS AND METHODS

### 2.1 Cancer cells and culture

The ATCC‐authenticated lung cancer cells (A549, NCI‐H460, NCI‐H1299, NCI‐H358, NCI‐H1650, and NCI‐H661) were purchased from the cell bank of Shanghai Institutes for Biological Sciences (Shanghai, China). A549 is a lung carcinoma cell line with the KRAS mutation. NCI‐H358 is a human non‐small cell lung cancer derived from the metastatic site (alveolus) and it expresses protein and RNA of lung surfactant‐associated protein A. NCI‐H1650 and NCI‐H460 are human lung carcinoma cells derived from pleural effusion; NCI‐H460 highly expresses p53 mRNA as compared to normal lung tissue. NCI‐H661 and NCI‐H1299 are human lung carcinoma cells derived from the lymph node. NCI‐H661 expresses p53 mRNA, whereas NCI‐H1299 lacks p53 mRNA and protein expression. SPC‐A1 is lung adenocarcinoma cells with high expression of surfactant‐associated protein A. HBE135‐E6E7 is used as a normal bronchial epithelium cell line. All cell lines were cultured in 10% FBS RPMI 1640 (Biowest, France) with 5% CO_2_ at 37°C. The cells at an exponential rate growth were used in our study.

### 2.2 Drug preparation

Chopped acRoots were mixed in 10‐fold double distilled water and heated to 100°C for 1 h. After two cycles of decoction, the concentration of 1 g/mL was ready to use.^2^

### 2.3 Cell screening study

NCI‐H1299, A549, NCI‐H460, SPC‐A1, NCI‐H358, HBE135‐E6E7, NCI‐H1650, and NCI‐H661(10^3^/well) were seeded in 96‐well plates with growth media. After attachment, cells were dealt with the acRoots at doses of 1, 5, 10, 30, 50, or 100 mg/mL for three time points (24, 48, and 72 h). The proliferation of cell was measured by CCK8 kit (Dojindo, Japan) and the viable cells number was measured by FlexStation 3 multimode microplate reader (Molecular Devices, USA). The inhibitory specificity and pattern on lung cancer cells treated with acRoots were evaluated by comparing with endometrial adenocarcinoma cell (RL95‐2), HCC cell (97H, 97L, Huh7, SMMC‐7721, LM3, HCCC‐9810, HepG2, and Hep3B), cholangiocarcinoma cells (Huh28), normal hepatic cells (L‐02), pancreatic cancer cells (SW1990, Panc‐1, CFPAC, and Mia‐paca), pancreatic stellate cells (PSC), leukemia cells (Rajli, Meg), glioma cells (U251), umbilical vein endothelial cells (HUVEC), prostatic cancer cells (Du145), breast cancer cells (MDA‐MB‐231 and MCF‐7), esophagus cancer cells (TE‐1 and Eca109), gastric cancer cells (AGS, MGC803, and SGC‐7901), and colon cancer cells (Caco‐2, SW480, HT‐29, RKO), as detailed in Table S1.

### 2.4 Measurement of transcriptional profiles

After cell screening study, H1299 cells lacking expression of p53 protein were selected as acRoots‐sensitive cells and H460 with easily detectable p53 mRNA as cells less sensitive to acRoots. Microarray experiments were performed as described previously.^4^

### 2.5 Bioinformatics analysis of hierarchical clustering and pathway networks

Hierarchical clustering was done by using TM4‐MEV (Multi Experiment Viewer, Dan‐Farber Cancer Institute, Boston, MA, USA) as described previously.^5^ Data were normalized as described previously^6^ for direct use. The normalized data then produced a heat map and hierarchal clustering by R language. KEGG pathway analyses were performed to identify the specific signaling pathway.^7^

### 2.6 Validation of selected acRoots‐associated target genes

After analyzed the microarray result, the selected acRoots‐associated target genes were validated by measuring gene expression of lung cancer cells by treating with vehicle or different doses of acRoots as well as in lung tissues harvested from patients with lung cancer. For validation of lung cancer cells, A549, H460, H1299, H358, H1650, H661, SPC‐A1, and HBE135‐E6E7 (5 × 10^4^/well) were seeded in well plates. After attachment, cells were treated with vehicle or acRoots. Then, TRIzol reagent (Invitrogen, USA) was used to collect the total RNA. Complementary DNA was composed using reverse transcription reagent kit (TaKaRa, Japan). Real‐time PCR was tested with primers (sequences were listed in Table S2) and SYBR (TaKaRa, Japan). The amplification of mRNA was detected by Q5 real‐time PCR System (Life Technology, USA). The relative expression of gene was normalized against the internal reference gene GAPDH. All real‐time PCRs were tested six times, and the relative fold of gene expression to untreated controls was exhibited.

For lung cancer tissues validation, human lung cancer samples and adjacent normal tissues were obtained from eight patients with lung cancer who were operated between July 2013 and September 2013 at the Department of Thoracic Surgery, Zhongshan Hospital, Fudan University. About 20‐mg freshly isolated tissues were lysated in TRIzol reagent (Invitrogen, USA). Total RNA was extracted according to the manufacturer's instruction. HSPA6 was measured according to the lung cancer cells validation by real‐time PCR. The experiment was approved by the Research Ethics Committee of Zhongshan Hospital, Fudan University.

### 2.7 HSPA6 RNA interference

Cell*^HSPA6−^* was developed using siRNA duplexes (GenePharma, China). Cells were incubated in well plate (10^5^ cells per well). After attachment, they were transfected with SiRNA HSPA6 primer (GCACAGGUAAGGCUAACAATT, UUGUUAGCCUUACCUGUGCTT) as well as negative control (UUCUCCGAACGUGUCACGUTT, ACGUGACACGUUCGGAGAATT) using Lipofectamine 2000 (Invitrogen, USA) following the manufacturer's direction. After incubation for 6 h, the media was discarded and cells were cultured in 10% FBS RPMI 1640 with different treatment.

### 2.8 Dynamic measurements of cell proliferation

The continuous dynamic cell proliferation information was recorded and analyzed by Cell‐IQ (Chip‐Man Technologies, Tampere, Finland). Cell proliferation rate of each point was calculated using the formula *N* = [*N*(*T_n_*) – *N*(*T*_0_)]/*N*(*T*_0_).

### 2.9 Apoptosis assay

Lung cancer cell apoptosis was measured using the Annexin V–FITC Apoptosis Detection Kit (BD Bioscience, USA), following the staining procedure as per manufacturer's protocol. Briefly, 10^5^ cells after single or combined drug treatment were washed with PBS and resuspended in binding buffer with Annexin V/propidium iodide staining. After staining, cells were detected and analyzed by flow cytometry (BD Bioscience). Approximately 20 000 cells were collected.

### 2.10 Statistical analysis

All analyses were performed with SPSS software version 13.0 (SPSS Inc., Chicago, USA). Data were exhibited as mean ± SD/SE. Normality was assessed by Shapiro‐Wilk *W* test. One‐way ANOVA was used to measure statistical differences between different groups, and Student's *t*‐test with one way and two tails and Wilcoxon rank‐sum (Mann‐Whitney) test were used to measure differences between two groups. *P*‐values less than 0.05 were considered statistically significant.

## 3 RESULTS

**FIGURE 1:**
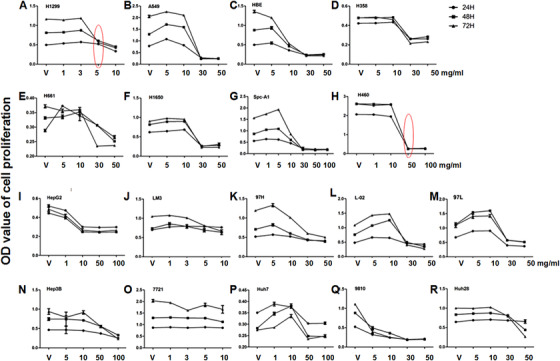
Inhibitory effects of acRoots in proliferation of cancer cells 24, 48, and 72 h after treatment with acRoots at doses of 1, 3, 5, 6, 10, 30, 50, and 100 mg/mL. Cancer cells mainly include lung cancer cells (H1299 [A], A549 [B], NCI‐H460 [C], NCI‐H358 [D], NCI‐H661 [E], NCI‐H1650 [F], Spc‐A1 [G], and H460 [H]), hepatocellular carcinoma cells (HepG2 [I], LM3 [J], 97H [K], 97L [M], Hep3B [N], 7721 [O], Huh7 [P], and 9810 [Q]), normal hepatic cells (L‐02 [L]), and cholangiocarcinoma cells (Huh28 [R]). The red circle stands for the lowest doses of acRoots with significant inhibition (*P* < 0.05 or less)

**FIGURE 2:**
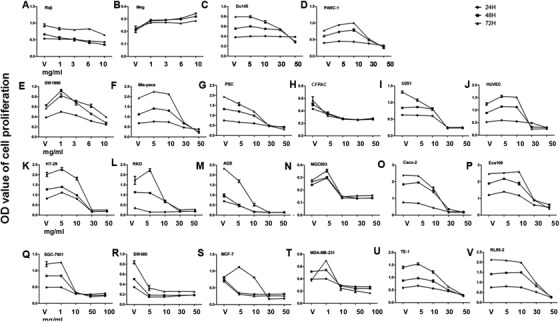
Inhibitory effects of acRoots in proliferation of cancer cells 24, 48, and 72 h after treatment with acRoots at doses of 1, 3, 5, 6, 10, 30, 50, and 100 mg/mL. Cancer cells mainly include leukemia cells (Raji [A] and Meg [B]), prostatic cancer cells (Du145 [C]), pancreatic cancer cells (Panc‐1 [D], SW1990 [E], Mia‐paca [F], and CFPAC [H]), pancreatic stellate cells (PSC [G]), glioma cells (U251 [I]), umbilical vein endothelial cells (HUVEC [J]), colon cancer cells (HT‐29 [K], RKO [L], Caco‐2 [O], and SW480 [R]), gastric cancer cells (AGS [M], MGC803 [N], and SGC‐7901 [Q]), breast cancer cells (MCF‐7 [S] and MDA‐MB‐231 [T]), esophagus cancer cells (TE‐1 [U] and Eca109 [P]), and endometrial adenocarcinoma cell (RL95‐2 [V]).

Of eight lung cancer cell lines, acRoots significantly stunted cell proliferation of H1299 at 5 mg/mL (Figure 1A); HBE (Figure 1C) and Spc‐A1 (Figure 1G) at 10 mg/mL; A549 (Figure 1B), H358 (Figure 1D), H661 (Figure 1E), and H1650 (Figure 1F) at 30 mg/mL; as well as H460 at 50 mg/mL. Of those cells, H1299 cells were selected as the sensitive cells and H460 as the less‐sensitive cells for the further validation. We also compared the A549 cell line with A549 p53 knockout cell line, and results showed that p53 knockout was more sensitive to acRoots treatment (Figure S3). To compare HCC cells, cholangiocarcinoma cells, and normal hepatic cells (Table S1), we found the significant inhibition in HepG2 (Figure 1I) and HCCC‐9810 (Figure 1Q) at 5 mg/mL; 97H (Figure 1K), L‐02 (Figure 1L), and 97L (Figure 1M) at 30 mg/mL; and Hep3B (Figure 1N) and Huh7 (Figure 1P) at 50 mg/mL and no effects in LM3 (Figure 1J) and SMMC‐7721 (Figure 1O). To compare with other cancer cells (Table S1), we found significant inhibitory effects in Rajli (Figure 2A), SW1990 (Figure 2E), CFPAC (Figure 2H), SW480 (Figure 2R), SGC‐7901 (Figure 2Q), MDA‐MB‐231 (Figure 2T), MGC803 (Figure 2N) at 5‐10 mg/mL; Du145 (Figure 2C), PANC‐1 (Figure 2D), and MCF‐7 (Figure 2S) at 30 mg/mL after 72 h; and Mia‐paca (Figure 2F), PSC (Figure 2G), U251 (Figure 2I), HUVEC (Figure 2J), HT‐29 (Figure 2K), Eca109 (Figure 2P), TE‐1 (Figure 2U), and RL95‐2 (Figure 2V) at 30 mg/mL during measurement, whereas there were no effects in Meg (Figure 2B).

**FIGURE 3:**
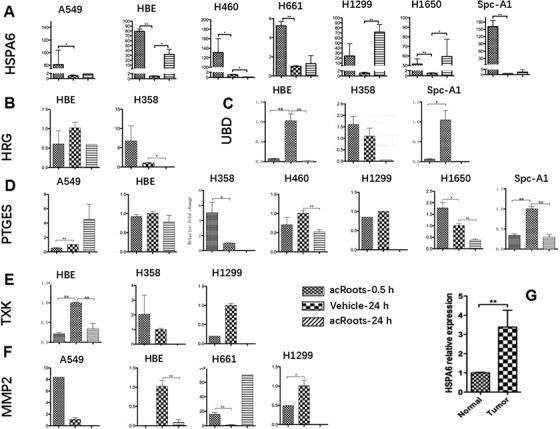
Selected sensitivity‐associated targets were validated in A549, H460, H1299, H358, H1650, H661, SPC‐A1, and HBE 0.5 and 24 h after treatment with vehicle or acRoots. A, heat shock 70 kDa protein 6 (HSPA6); B, histidine‐rich glycoprotein (HRG); C, ubiquitin D (UBD); D, prostaglandin E synthase (PTGES); E, tyrosine kinase (TXK); F, matrix metallopeptidase 2 (MMP2). G, Expression of HSPA6 was validated in lung adenocarcinoma tissue (tumor) and adjunct normal tissue (normal)

**FIGURE 4:**
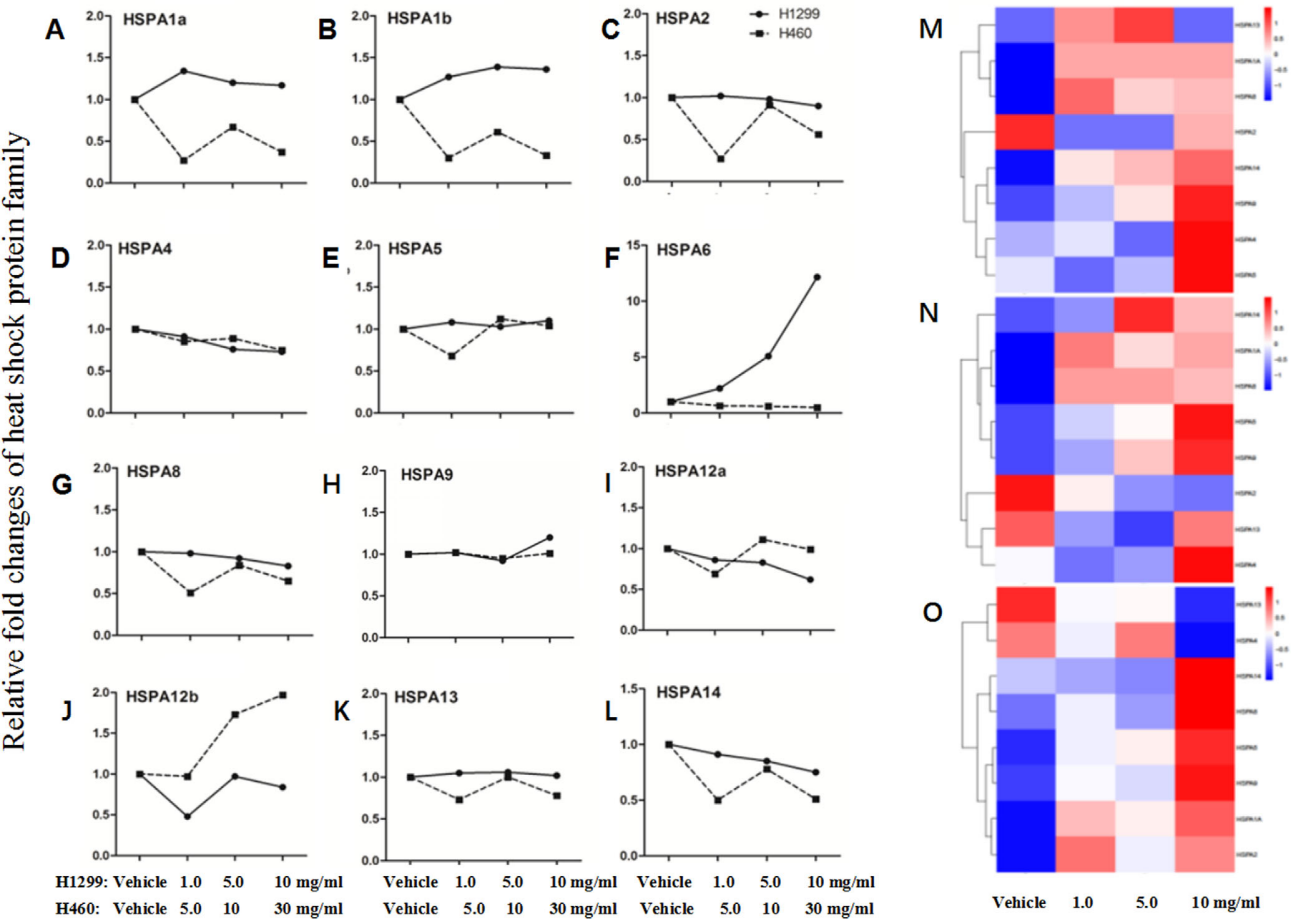
Gene expression of HSPA family members 24 h after vehicle or acRoots at doses of 1.0, 5.0, and 10 mg/mL (H1299) and 5.0, 10, and 30 mg/mL (H460), including HSPA1a (A), HSPA1b (B), HSPA2 (C), HSPA4 (D), HSPA5 (E), HSPA6 (F), HSPA8 (G), HSPA9 (H), HSPA12a (I), HSPA12b (J), HSPA13 (K), and HSPA14 (L). Protein expression of HSPA family members 24 h (M), 48 h (N), and 72 h (O) after vehicle or acRoots at a dose of 1.0, 5.0, and 10 mg/mL (H1299), including HSPA1a, HSPA2, HSPA4, HSPA5, HSPA8, HSPA9, HSPA13, and HSPA14

We measured transcriptional profiles of acRoots‐sensitive and less‐sensitive cells (H1299 and H460) 24 h after treatment with acRoots at doses of 1, 5, and 10 mg/mL (H1299) and 5, 10, and 30 mg/mL (H460) and identified up‐ and downregulated gene expression twofold above cells treated with vehicle, as detailed in Figure S1 and our previous publication.^8^ Of those identified target genes, we selected HSPA6 (Figure 3A), histidine‐rich glycoprotein (HRG) (Figure 3B), ubiquitin D (Figure 3C), prostaglandin E synthase (PTGES) (Figure 3D), tyrosine kinase (TXK) (Figure 3E), and MMP2 (Figure 3F) and measured those gene expressions in eight lung cancer cells 48 h after treatment with acRoots at 10 mg/mL (Figure 3). The expression of HSPA6 gene apparently increased in HBE, H1299, and H1650 cells (*P* < .05; Figure 4A), whereas it decreased in H460 (*P* < .05). We furthermore evaluated gene expression of HSPA6 in patients with lung adenocarcinoma (Table 1) and found that HSPA6 gene overexpressed in lung adenocarcinoma tissue, as compared to the adjuvant normal tissue (*P* < 0.01; Figure 3G). Those selected target genes had no significant correlation with overall survival rates in patients with lung squamous cell carcinoma (Figure S2A) and lung adenocarcinoma (Figure S2B), except HRG in lung adenocarcinoma.

**Table 1:** Clinicopathologic characteristics in eight patients with non‐small cell lung cancer

Patient no.	Gender	Age	Cancer type	Differentiation	TTF‐1	NapsinA	HCK	CK7
1	Female	51	Adenocarcinoma	II‐III	80%+	100%+–++	100%+	100%++
2	Male	58	Adenocarcinoma	II	+	+	70%+	100%+++
3	Male	61	Adenocarcinoma	II	100%+	100%+	100%+–++	100%++
4	Female	55	Adenocarcinoma	II	100%+	100%+	70%+	100%+++
5	Female	60	Adenocarcinoma	II				
6	Female	56	Adenocarcinoma	II	100%++	100%++	20%+	100%+++
7	Female	56	Adenocarcinoma	II	+	++	+–++	
8	Female	62	Adenocarcinoma	II	90%+	100%++	50%+	100%+++

In order to understand biological activities of HSP family members, we measured gene expression of HSPA1a (Figure 4A), HSPA1b (Figure 4B), HSPA2 (Figure 4C), HSPA4 (Figure 4D), HSPA5 (Figure 4E), HSPA6 (Figure 4F), HSPA8 (Figure 4G), HSPA9 (Figure 4H), HSPA12a (Figure 4I), HSPA12b (Figure 4J), HSPA13 (Figure 4K), and HSPA14 (Figure 4L) in H1299 and H460 cells treated with acRoots at doses of 1, 5,10, and 30 mg/mL. The gene expression of HSPA1a and HASPA1b decreased and HSPA12b increased in H460. The expression of HSPA6 gene significantly increased about 15‐folds in H1299 cell higher than that in H460. We then measured the protein expression of HSP family members in H1299 at doses of 1, 5, and 10 mg/mL. After 24 h, HSPA1A, HSPA8, HSPA2, HSPA14, HSPA9, HSPA4, and HSPA5 were upregulated by acRoots treatment at a dose of 10 mg/mL (Figure 4M). After 48 h, HSPA14, HSPA1A, HSPA8, HSPA5, HSPA9, HSPA13, and HSPA4 were increased in protein level by acRoots treatment at a dose of 10 mg/mL (Figure 4N). After 72 h, HSPA14, HSPA8, HSPA5, HSPA9, HSPA1A, and HSPA2 were upregulated by acRoots treatment at a dose of 10 mg/mL on protein level (Figure 4O).

**FIGURE 5:**
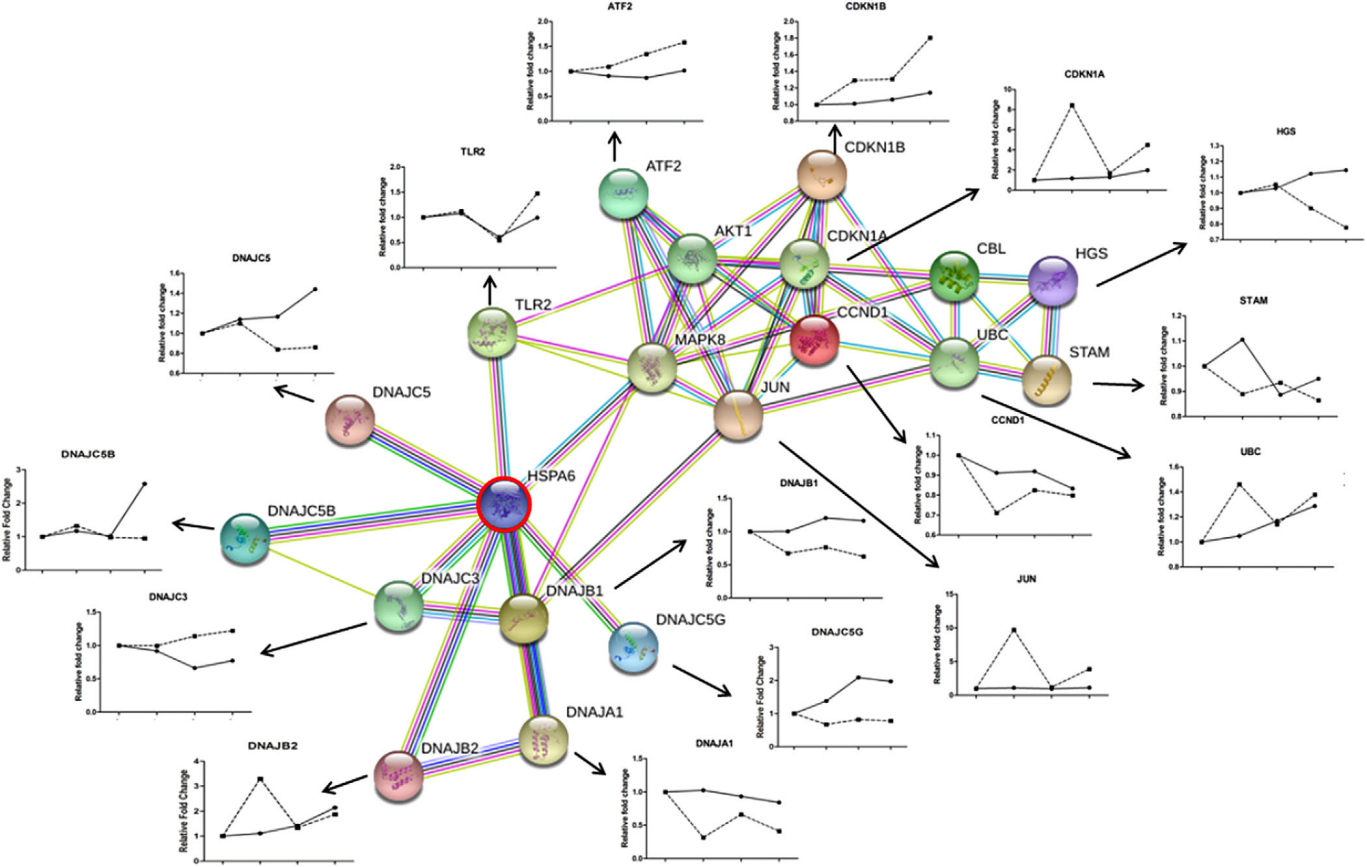
HSPA6‐dominated molecular networks and interactions with HSP40 family and other families 24 h after vehicle or acRoots at doses of 1.0, 5.0, and 10 mg/mL (H1299) and 5.0, 10, and 30 mg/mL (H460), including HSP40 family (DNAJB1, DNAJC3, DNAJC5B, DNAJC5B, DNAJB2, DNAJA1, and DNAJ5), ubiquitin D (UBD), cyclin‐dependent kinases inhibitor p16/CDKNs (CDKN1A and CDKN1B), cyclin D (CCND1), JUN, activating transcription factor 2 (ATF2), Toll‐like receptor 2 (TLR2), and human glutamine synthetase (HGS)

In HSPA6‐dominated signal pathway networks, alterations of HSPA6 in H1299 and H460 cells were positively correlated with changes of DNAJC5G, DNAJB1, DNAJC5B, DNAJC5, and human glutamine synthetase (HGS), whereas negatively with DNAJC3, activating transcription factor 2 (ATF2), and CDKN1B (Figure 5).

**FIGURE 6:**
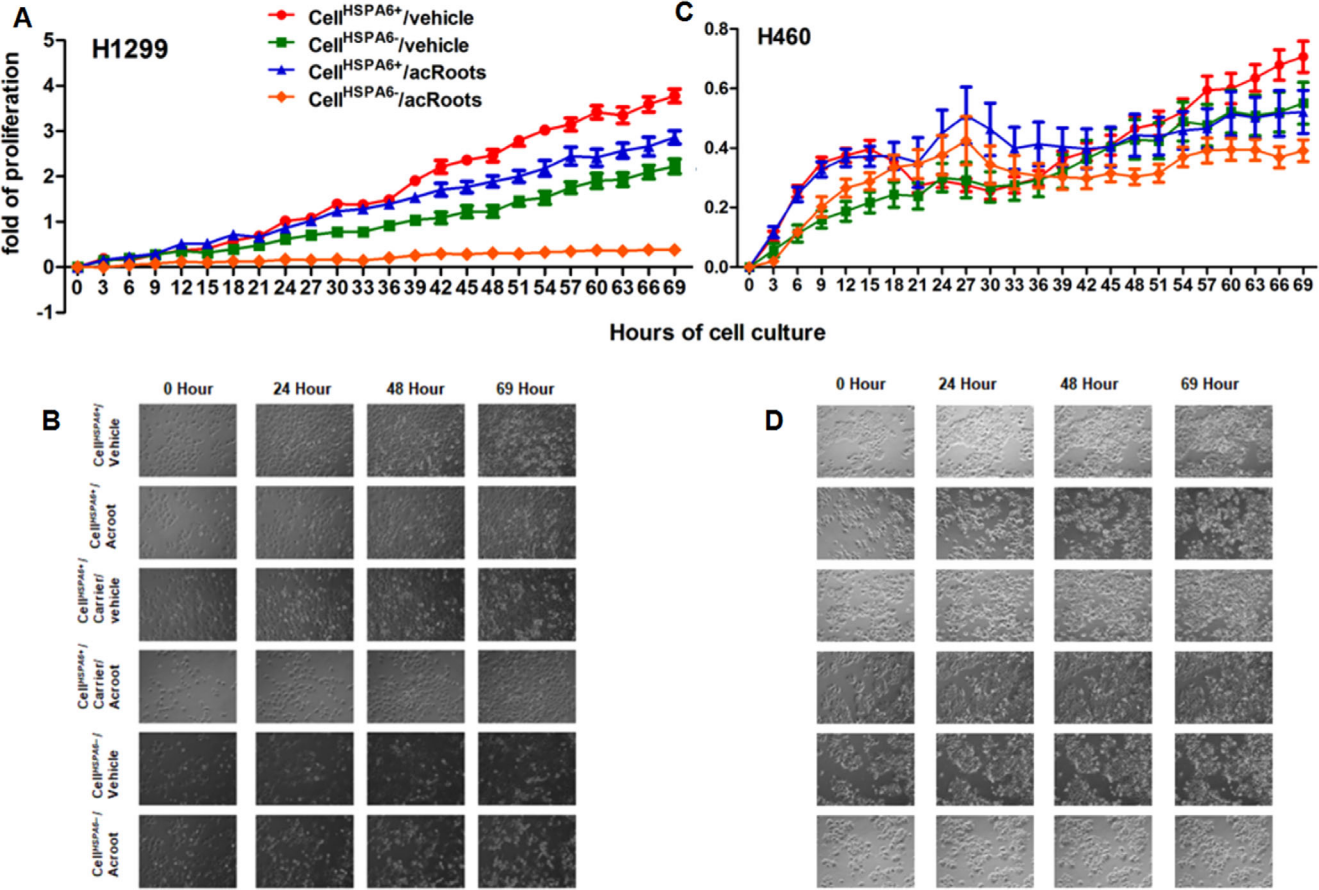
Dynamic proliferations of H1299 cells (A and B) and H460 cells (C and D) in cell*^HSPA6+^* or cell*^HSPA6−^* 72 h after treatment with vehicle or 5 mg/mL acRoots

**FIGURE 7:**
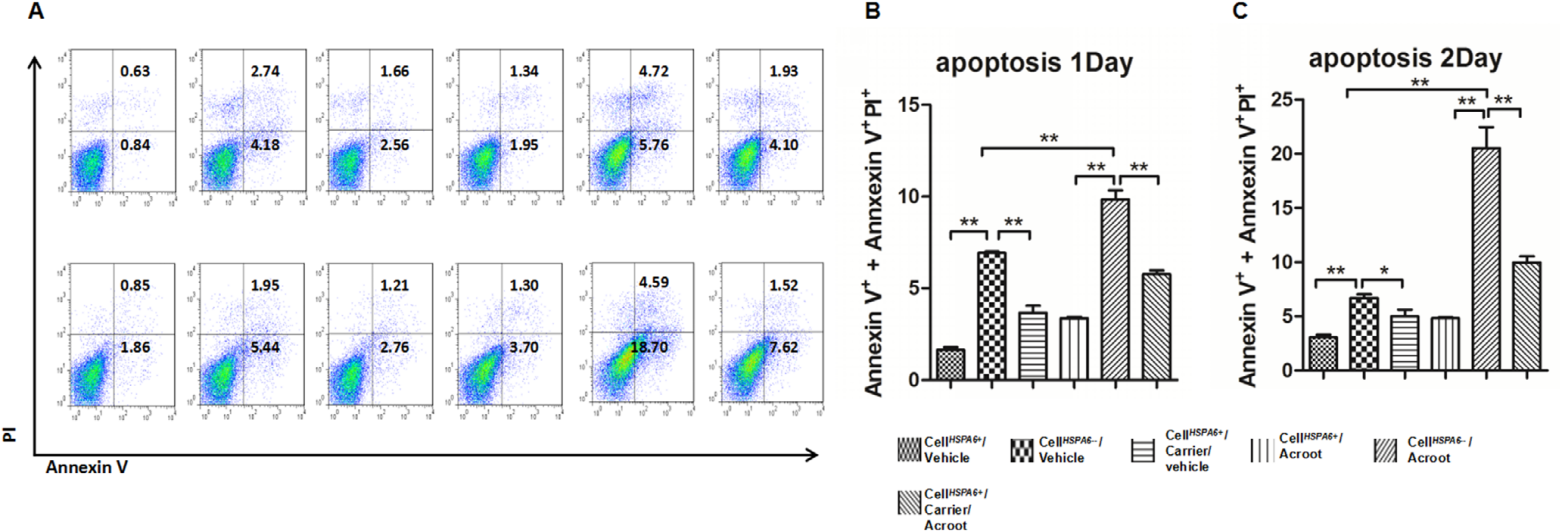
Expression of Annexin V in H1299 cells measured by flow cytometry (A) and apoptotic cell number on day 1 (B) and 2 (C) in cell*^HSPA6+^* or cell*^HSPA6−^* 72 h after treatment with vehicle or 5 mg/mL acRoots with or without carriers

In order to further evaluate the decisive role of HSPA6 in cell responses to acRoots, we dynamically measured proliferation of cell*^HSPA6−^* and cell*^HSPA6+^* with 72 h after treatment with acRoots and found that proliferation of H1299 cell*^HSPA6−^* treated with vehicle or acRoots was significantly lower than that of H1299 cell*^HSPA6+^* with vehicle or acRoots (*P* < 0.05; Figures 6A and 6B). The difference of cell proliferation in H1299 cell*^HSPA6−^* with vehicle and acRoots was significantly more than that in H1299 cell*^HSPA6+^* with vehicle and acRoots. We also noticed that the proliferation of H1299 cell*^HSPA6−^* with vehicle was significantly lower than that of H1299 cell*^HSPA6+^* with vehicle or acRoots. There was no significant difference of cell proliferation in H460 cell*^HSPA6+^* with vehicle and acRoots, in H460 cell*^HSPA6−^* with vehicle and acRoots, and between H460 cell*^HSPA6+^* and H460 cell*^HSPA6−^* (Figures 6C and 6D). The proliferation of H460 cell*^HSPA6+^* with acRoots significantly declined from 60 h, whereas the proliferation of H460 cell*^HSPA6−^* with acRoots declined from 40 h after cell culture. The results from flow cytometry (Figure 7A) demonstrated that acRoots significantly increased the number of apoptotic cells with or without HSPA6 day 1 (Figure 7B) and day 2 (Figure 7C) after treatment with vehicle or acRoots. We also noted that the number of apoptotic cells in H1299 cell*^HSPA6−^* with vehicle was higher than that in H1299 cell*^HSPA6+^* with vehicle and that the number of apoptotic cells in H1299 cell*^HSPA6−^* with acRoots was significantly higher than that in H1299 cell*^HSPA6−^* with vehicle and H1299 cell*^HSPA6+^* with vehicle and acRoots.

## 4 DISCUSSION

AcRoots as part of Chinese traditional medicine has been applied as tumor therapy for decades and was investigated as a tumor inhibitor in many tumors since the middle of the 20th century, including gastric cancer,^9^, ^10^ liver cancer,^2^, ^3^ and lung cancer.^1^, ^8^ Of lung cancer cells with various genetic backgrounds, we found that lung cancer cells without expression of p53 were about 10‐fold more sensitive to acRoots than cells with p53. Similar results were validated by the lung cancer cell A549 with or without p53 by Crispr‐Cas9. High mutation rates of p53 in lung cancer were associated with the development of acquired drug resistance, dependent on target specificities, mechanism‐specific signal pathways, drug chemical properties, and drug efficacies, but not on the size of molecules.^10^ p53‐mutation‐associated alterations of p53 function and regulation contribute to the development of chemoresistance and clinical phenomes of drug resistance.^11^ p53 mutation‐based drug resistance has been proposed as one of the criteria to design therapeutic strategy for complex cancer.^12^ Our study indicates that the sensitivity to acRoots of lung cancer cells seems to dependent on the degree of p53 mutation, because the other lung cancer cells, for example, A549, H358, H1650, H661, SPC‐A1, and HBE135‐E6E7 that contain intact or partial p53 at different degrees, had the sensitivity to acRoots between cells with p53 high expression and without p53. Another potential is that other factors may be involved in the sensitivity to drug of lung cancer cells.

The present study first compared inhibitory efficacy of acRoots in lung cancer cells with endometrial adenocarcinoma cells, HCC cells, cholangiocarcinoma cells, normal hepatic cells, pancreatic cancer cells, PSC, leukemia cells, glioma cells, umbilical vein endothelial cells, prostatic cancer cells, breast cancer cells, esophagus cancer cells, gastric cancer cells, and colon cancer cells. Of HCC cells, we noticed that the HCCC‐9810 cell driven from intrahepatic cholangiocarcinoma was the most sensitive to acRoots, like other drugs,^13^ although the genetic variation remains unclear, and that the Hep3B cell was the least sensitive. Human hepatoma Hep3B cells appeared as the least sensitive to acRoots and more sensitive to cell killing drugs like fenofibrate, probably associated with the closely related fibroblasts, the origin from more differentiated liver cells in hepatic lobule.^14^ We also found that inhibitory effects of acRoots varied among cancer cell types, although the exact mechanism is poorly understood. For example, acRoots inhibited the growth of leukemia cells Rajli, but not Meg cells. AcRoots showed obviously inhibitory effects in pancreatic cancer cells and PSC, colon cancer cells, and gastric cancer cells.

We selected HSPA6, HRG, PTGES, TXK, and MMP2 on the basis of the present study and previous findings.^8^ Of those factors, the expression of HSPA6 gene downregulated in less‐sensitive cells and upregulated in sensitive cells. As acRoots was sensitive to the p53^−^ cell lines, it indicated that acRoots‐treated HSPA6 upregulation might have some interaction with p53 or its related pathways. Of HSP family, proteins of the HSP90 (HSPC) family members were overexpressed in non‐small cell lung cancer cells and multiple inhibitors were developed for clinical trials.^15^ The expression and functional impact of the HSPA family member genes and proteins among subtypes of lung cancer remain poorly understood. The present study first demonstrated that HSPA6 gene was overexpressed in lung adenocarcinoma tissues. The HSPA6 gene is potentially to be a combined drug therapy target in lung cancer. HSPA6 (HSP70B’) is an inducible member of the HSPA (HSP70) family and it is present in the human genome, contributing to cell proliferation and biological response through stress‐sensitive sites with a disaggregation/refolding machine and transcriptional sites with perispeckles.^16^ Previous study evidenced that there was no clear correlation between protein expressions of HSPA family members (eg, HSPA1, 2, 5, and 8) and lung cancer cell susceptibility to cisplatin or between drug‐sensitive and nonsensitive cells.^17^ In this study, we evaluated the gene expression of 12 HSPA family members (eg, 1a, 1b, 2, 4, 5, 6, 8, 9, 12a, 12b, 13, and 14) in acRoots‐sensitive and less‐sensitive cells and found that the expression of HSPA1a, 1b, and 6 genes increased in sensitive cells and declined in less‐sensitive cells.

**FIGURE 8:**
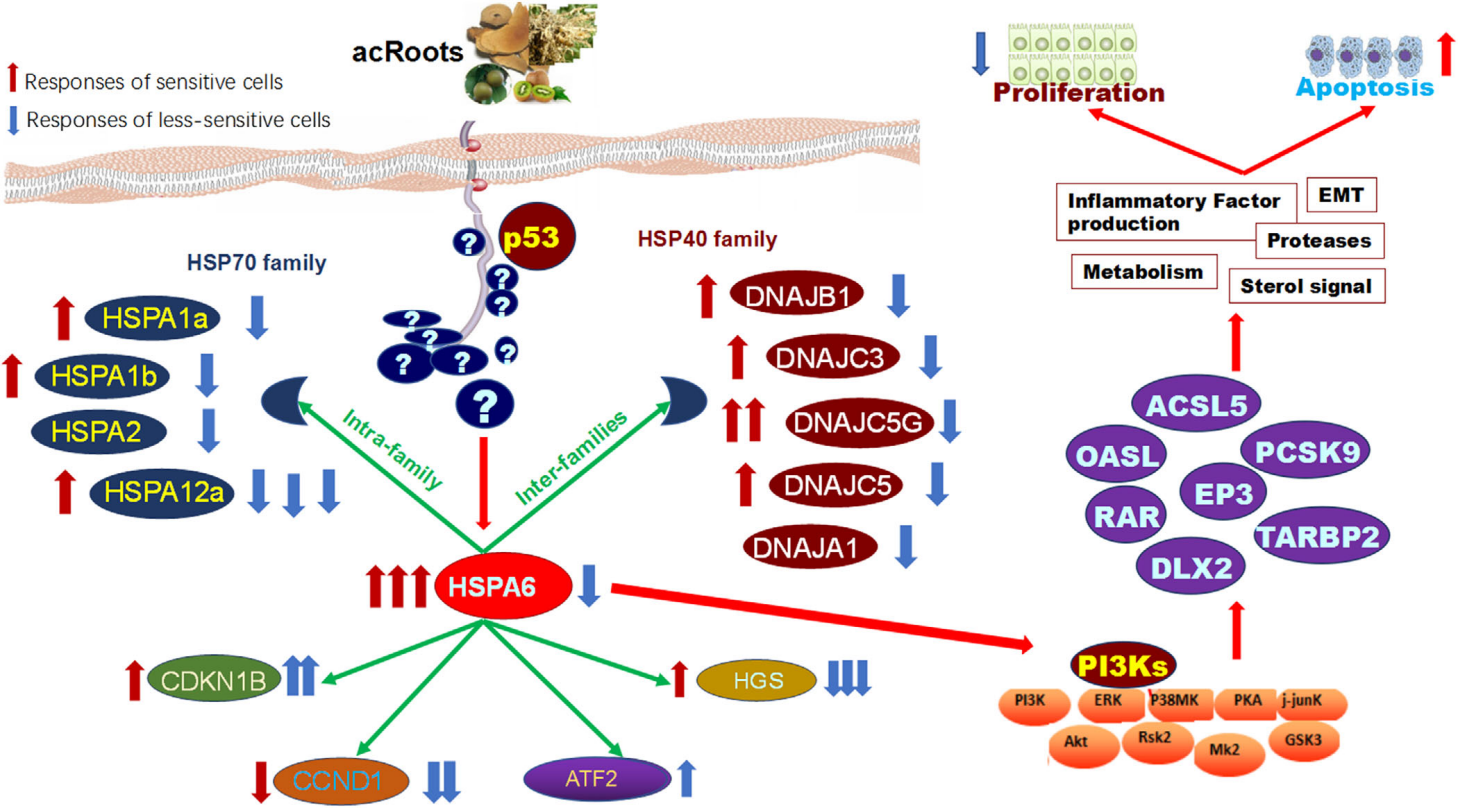
Summary of proposed molecular mechanisms by which acRoots inhibited lung cancer cell proliferation by altering the number of apoptotic cells in p53‐dependent or independent pathways through activities of HSPA6, although the detail factors between acRoots and HSPA6 remain unknown as marked question marks. HSPA6 may interact with HSPA family members, HSP40 family, and other families in acRoots‐sensitive (red arrows) and less‐sensitive cells (blue arrows). Through the interactions, HSPA6 may activate other transcriptional factors such as long‐chain acyl‐Co A synthetase, OASL, prostaglandin E receptor 3 (EP3), retinoic acid receptor (RAR), distal‐less homeobox 2 (DLX2), TARBP2 through PI3Ks, and other signal factors, leading to inflammatory factor overproduction, epithelial‐mesenchymal transition (EMT) activation, metabolism abnormalities, protease overactivations, and sterol signal disorders

Of HSPA family members, the gene expression of HSPA6 or HSPA12b increased more obviously in sensitive or less‐sensitive cells, respectively. Those data indicate that some of HSPA family members may have coordinated functions of positive or negative interregulations in response to acRoots, as explained in Figure 8. The coordinated function between HSPA1a and HSPA6 was found to control cell survivals in responses to the physiological and pathophysiological conditions.^18^ The present data imply that acRoots‐induced transcriptional expression of HSPA1a and HSPA1b genes altered parallelly with HSPA6 in a dose‐dependent pattern. In addition to the intra‐HSPA family co‐regulation, there is also an inter‐HSP family regulatory network or HSPA‐dominated molecular network (Figure 8). Among small HSPs (HSPB), HSP40 (DNAJ), HSP60, HSP70 (HSPA), HSP90 (HSPC), and HSP100 (HSPH) families, acRoots‐induced HSPA6 alternations may interact with HSP40 family members, for example, DNAJB1, DNAJC3, DNAJC5G, DNAJC5, and DNAJA1. Within HSPA6‐dominated dynamic molecular networks in response to acRoots, HGS was positively associated with HSPA6, whereas the cyclin‐dependent kinase inhibitor 1B and ATF2 were negatively associated with HSPA6.

The present study proposed the HSPA6‐dominated transcriptional signals as the part of molecular mechanisms of lung cancer cell sensitivity to acRoots on the basis of acRoots‐altered profiles of transcriptional factors. The important role of HSPA6 in cell sensitivity to acRoots was furthermore evidenced by the deletion of HSPA6. We found that the deletion of HSPA6 reduced the capacity of cell survival and proliferation, which varied among cells. HSPA6 is a decisive factor in regulation of cell maximal survival, and downregulation of HSPA6 expression reduced the cell proliferation during stress challenge.^18^ Our data showed that acRoots induced the inconsistent and varied expression of HSP elements between families and between members in a family. This is supported by the previous finding that heat shock could reduce the expression of HSPA6 rather than other HSP members.^19^ To our surprise, the endogenous HSPA6 increased in acRoots‐sensitive cells, whereas both sensitive and less‐sensitive cells became more sensitive in HSPA6 deletion through the increase in apoptotic cell number. It seems that HSPA6 contributes to cell sensitivity to acRoots in various cell types through different signaling pathways, and exact mechanisms need to be further investigated. For example, TNF‐α‐induced protein 3‐interacting protein 1 could regulate HSPA6 probably through unexpected factors independent of the capacity of binding sites in the HSPA6 promoter.^19^ The cell sensitivity to acRoots may attribute to the interaction of HPSA6 with HSPA family and HSP family members as well as other families. Our previous studies evidenced that other factors such as long‐chain acyl‐Co A synthetase, OASL, PCSK9, EP3, retinoic acid receptor, and distal‐less homeobox 2 could also play critical roles in cancer cell sensitivity to acRoots,^1^, ^2^, ^3^, ^10^, ^20^ as explained in Figure 8. And PI3K pathway was important in the regulation of acRoots treatment.^1^ Then, it is possible that HPSA6 may regulate cell sensitivity to acRoots mainly through p53‐dependent pathways, probably leading the secondary changes in chemical structures and properties of PI3K subunit proteins or in interactions between p53 and PI3K isoform genes.^21^, ^22^

In conclusion, the present study selected acRoots‐sensitive and less‐sensitive lung cancer cells and found that the sensitivity was associated with the appearance of p53. Inhibitory effects of acRoots were noticed in multiple cancer types and varied with genetic backgrounds. HSPA6 was identified as a critical factor in regulating cell sensitivity probably through the interaction with intra‐HSPA family members, inter‐HSP family members, and other families. The degree of cell sensitivity to acRoots increased in both sensitive and less‐sensitive cells after deletion of HSPA6 genes. Thus, our data indicate that HSPA6 and HSPA6‐dominated molecular network can be an alternative to modify cell sensitivity to drugs.

## Supporting information

Supporting information

Supporting information

Supporting information

Supporting information
