# Supplementary figures and images for: Regulatory roles of HSPA6 in Actinidia chinensis Planch. root extract (acRoots)‐inhibited lung cancer proliferation
Source: Clin Transl Med. 2020 Jun 5;10(2):e46. doi: 10.1002/ctm2.46 (PMC7403824; doi:10.1002/ctm2.46)

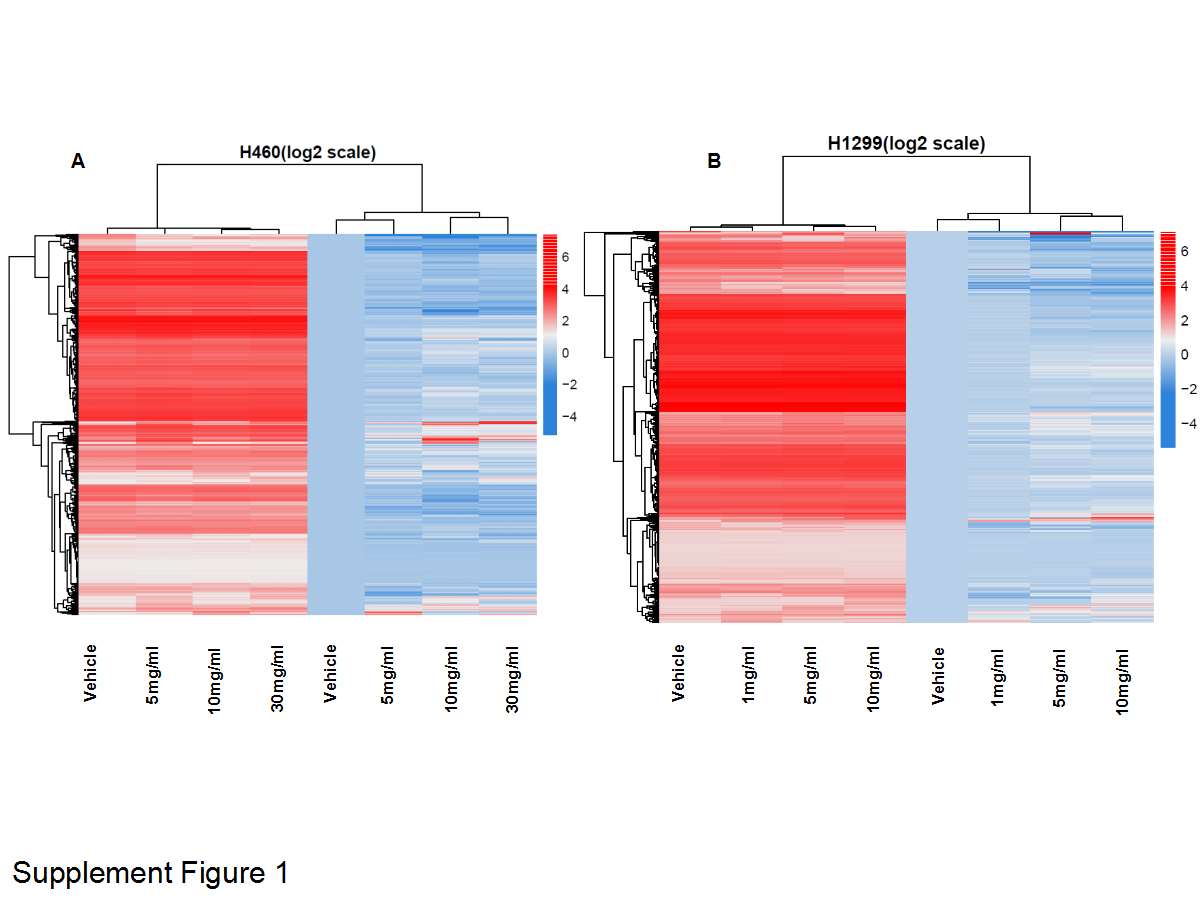

Supplement: Supplementary file 1 — Supporting information [file CTM2-10-e46-s001.tif]

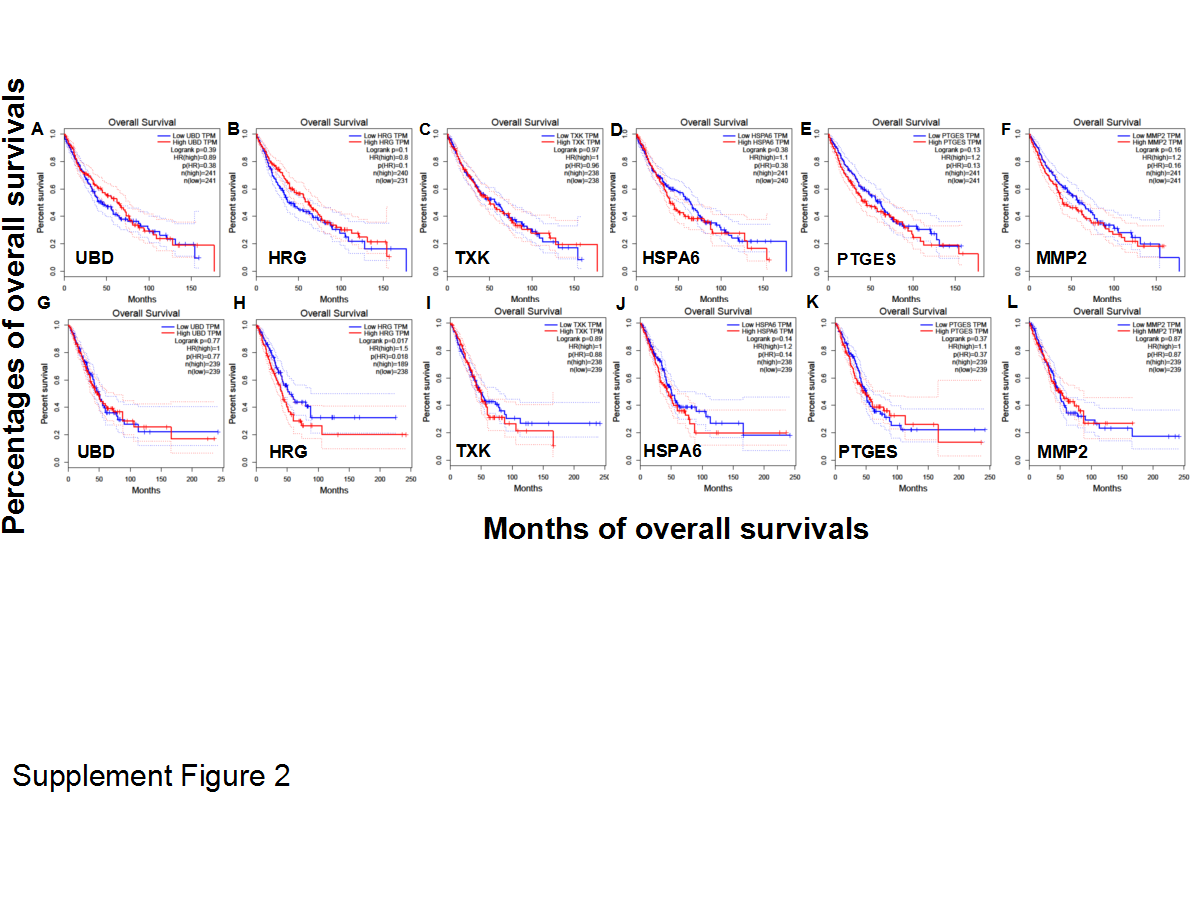

Supplement: Supplementary file 2 — Supporting information [file CTM2-10-e46-s002.tif]

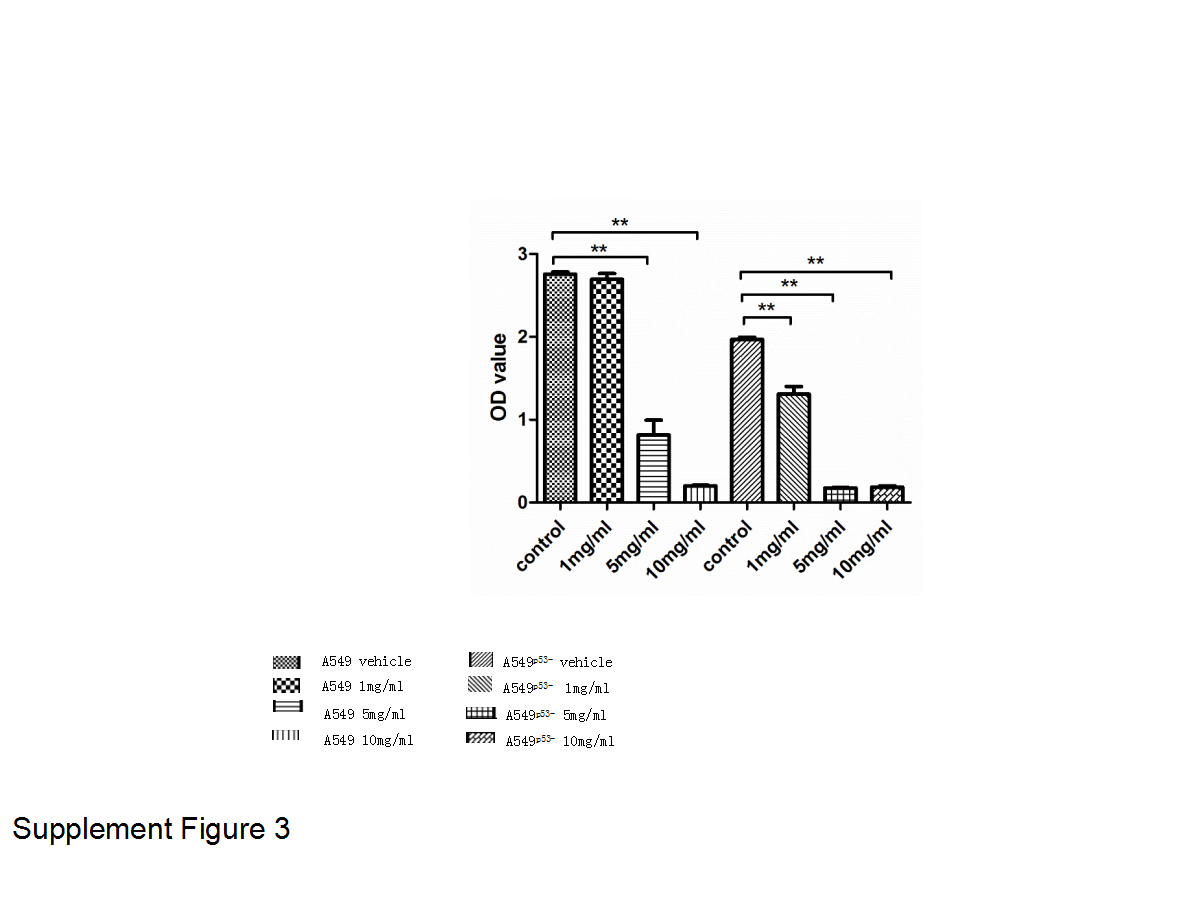

Supplement: Supplementary file 3 — Supporting information [file CTM2-10-e46-s003.tif]
