# Supplementary material for: Regulatory roles of HSPA6 in Actinidia chinensis Planch. root extract (acRoots)‐inhibited lung cancer proliferation
Source: Clin Transl Med. 2020 Jun 5;10(2):e46. doi: 10.1002/ctm2.46 (PMC7403824; doi:10.1002/ctm2.46)
Supplement: Supplementary file 4 — Supporting information [file CTM2-10-e46-s004.docx]

**Supplemental Table1：Information of the cell lines in acRoots treatment (Data is from ATCC website)**

| **Cell line name** | **Cancer type** | **Source** | **Specificity** |
| --- | --- | --- | --- |
| H661 | Large cell lung cancer | Derived from metastatic site: lymph node | The cells express easily detectable p53 mRNA at levels comparable to normal lung tissue, and exhibit no gross structural DNA abnormalities. |
| H460 | Large cell lung cancer | Lung: pleural effusion | The cells express easily detectable p53 mRNA at levels comparable to normal lung tissue, and exhibit no gross structural DNA abnormalities.  The cells stain positively for keratin and vimentin but are negative for neurofilament triplet protein. |
| H358 | Non-small cell lung cancer | Derived from metastatic site: alveolus | The cells expressed protein and RNA of SP-A, the major lung surfactant associated protein.  SP-B and SP-C RNA was not expressed. |
| H1650 | Bronchoalveolar carcinoma | Derived from metastatic site: pleural effusion | The cells have a mutation of EML3 SGIP1 et al. |
| H1299 | Non-small cell lung cancer | Derived from metastatic site: lymph node | The cells have a homozygous partial deletion of the p53 protein, and lack expression of p53 protein. |
| A549 | Lung adenocarcinoma | Lung | This cell line has been tested for the KRAS mutation (p.G12S c.34G>A). |
| HBE | Normal human airway epithelial cells | Human normal respiratory tract | Adherent growth |
| Spc-A1 | Lung adenocarcinoma | Lung | Adherent growth |
| RL95-2 | Endometrial carcinoma | Uterus; endometrium | The cells possess alpha keratin, well defined junctional complexes, tonofilaments and surface microvilli. |
| 97H | Hepatocellular carcinoma | Liver | High metastasis. |
| 97L | Hepatocellular carcinoma | Liver | Low metastasis. |
| Huh7 | Hepatocellular carcinoma | Liver | Adherent growth |
| SMMC-7721 | Hepatocellular carcinoma | Liver | The cells are positive of AFP and lack the expression of LFIRE-1/HFREP-1 mRNA. |
| LM3 | Hepatocellular carcinoma | Liver | High metastasis. |
| HCCC-9810 | [Intrahepatic](javascript:;) [cholangiocarcinoma](javascript:;) | Liver | The cells secret low level of AFP、CEAand CA19-9. |
| HepG2 | Hepatocellular carcinoma | Liver | The cells express 3-hydroxy-3-methylglutaryl-CoA reductase and hepatic triglyceride lipase activities. |
| Hep3B | Hepatocellular carcinoma | Liver | The cells express alpha-fetoprotein, BHsAg, alphal-antitrypsin and C3. |
| Huh28 | [Intrahepatic](javascript:;) [cholangiocarcinoma](javascript:;) | Liver | Adherent growth |
| L-02 | Normal hepatic cell | Liver | Adherent growth |
| SW1990 | Pancreatic adenocarcinoma | Derived from metastatic site: spleen | Antigen expression: Blood Type A; Rh ^+^. |
| Panc-1 | Pancreatic epithelioid carcinoma | Pancreas/duct | Growth is inhibited by 1 unit/mL L-asparaginase.  The cells will grow in soft agar. |
| CFPAC | Pancreatic carcinoma | Pancreas; derived from metastatic: liver | The cells express CFTR. |
| Mia-paca | Pancreatic carcinoma | Pancreas | The cells have been tested for KRAS mutation (p.G12C c.34G>T). |
| PSC | Pancreatic stellate cell | Pancreatic stroma | The cells express TGF-β,IL-6,FGF2 and TGF-α |
| Raji | Burkitt's lymphoma cell | B lymphocyte | These cells are EBNA positive.  The cells are partially resistant to poliovirus and vesicular stomatitis viruses. |
| Meg | Chronic myelogenous leukemia cell | Bone marrow | The cells are positive for cytoplasmic Factor VIII and surface GPIIb/IIIa, periodic acid - Schiff (PAS) reaction, alpha naphthyl acetate esterase and acid phosphatase.  They are negative for myeloperoxidase, alpha naphthyl butyrate esterase, naphthol AS-D chloroacetate esterase and alkaline phosphatase. |
| U251 | [Glioma](javascript:;) cell | Glioma | Adherent growth |
| HUVEC | Primary Umbilical Vein Endothelial Cells | Umbilical | Adherent growth |
| Du145 | Prostatic carcinoma | Derived from metastatic site: brain | The line is not detectably hormone sensitive, is only weakly positive for acid phosphatase and isolated cells form colonies in soft agar. The cells do not express prostate antigen. |
| MCF-7 | Breast ductal carcinoma | Mammary gland, breast; derived from metastatic site: pleural effusion | The cells express the WNT7B oncogene. |
| MDA-MB-231 | Breast adenocarcinoma | Mammary gland/breast; derived from metastatic site: pleural effusion | The cells express the WNT7B oncogene. |
| TE-1 | [esophagus](javascript:;) [cancer](javascript:;) | [esophagus](javascript:;) | Adherent growth |
| Eca109 | [esophagus](javascript:;) [cancer](javascript:;) | [esophagus](javascript:;) | Adherent growth |
| AGS | Gastric adenocarcinoma | Stomach | Adherent growth |
| MGC803 | Gastric adenocarcinoma | Stomach | Adherent growth |
| SGC-7901 | Gastric adenocarcinoma | Stomach | Adherent growth |
| Caco-2 | Colorectal adenocarcinoma | Colon | Caco-2 cells express retinoic acid binding protein I and retinol binding protein II. |
| SW480 | Colorectal adenocarcinoma | Colon | The cells express elevated levels of p53 protein.  The line is positive for expression of c-myc, K-ras, H-ras, N-ras, myb, sis and fos oncogenes.  N-myc oncogene expression was not detected. |
| HT-29 | Colorectal adenocarcinoma | Colon | The cells express urokinase receptors, but do not have detectable plasminogen activator activity. HT-29 cells are negative for CD4, but there is cell surface expression of galactose ceramide (a possible alternative receptor for HIV). |
| RKO | Colon carcinoma | Colon | RKO cells contain wild-type p53 but lack endogenous human thyroid receptor nuclear receptor (h-TRbeta1). |

**Supplement table 2: Sequences for gene detection.**

| **Gene** | **Sequences** |
| --- | --- |
| HSPA6 | Forward: GCCCTGAACCCCCACAACAC  Reverse: CCTCGGCCGTCTCCTTCATCT |
| HRG | Forward: TCACATTGCAGTATTCGTGTGC  Reverse: CATCCCGTCGCCTTTTATTGA |
| UBD | Forward: CCGTTCCGAGGAATGGGATTT  Reverse: GCCATAAGATGAGAGGCTTCTCC |
| PTGES | Forward: TCCTAACCCTTTTGTCGCCTG  Reverse: CGCTTCCCAGAGGATCTGC |
| TXK | Forward: CATCCAGTCGGTTTTCTGTTGC  Reverse: TGCGACGCTGGGTGTATTTT |
| MMP2 | Forward: TACAGGATCATTGGCTACACACC  Reverse: GGTCACATCGCTCCAGACT |
| GAPDH | Forward: AGAAGGCTGGGGCTCATTT  Reverse: AGGGGCCATCCACAGTCTTC |
